# ERC/mesothelin is expressed in human gastric cancer tissues and cell lines

**DOI:** 10.3892/or.2013.2803

**Published:** 2013-10-22

**Authors:** TOMOAKI ITO, KAZUNORI KAJINO, MASAAKI ABE, KOICHI SATO, HIROSHI MAEKAWA, MUTSUMI SAKURADA, HAJIME ORITA, RYO WADA, YOSHIAKI KAJIYAMA, OKIO HINO

**Affiliations:** 1 Department of Surgery, Juntendo Shizuoka Hospital, Juntendo University School of Medicine, Bunkyo-ku, Tokyo, Japan; 2 Department of Pathology and Oncology, Juntendo University School of Medicine, Bunkyo-ku, Tokyo, Japan; 3 Department of Pathology, Juntendo Shizuoka Hospital, Juntendo University School of Medicine, Bunkyo-ku, Tokyo, Japan; 4 Department of Esophageal and Gastroenterological Surgery, Juntendo University School of Medicine, Bunkyo-ku, Tokyo, Japan

**Keywords:** mesothelin, ERC/mesothelin, C-ERC/mesothelin, N-ERC/mesothelin, gastric cancer

## Abstract

ERC/mesothelin is expressed in mesothelioma and other malignancies. The ERC/mesothelin gene (*MSLN*) encodes a 71-kDa precursor protein, which is cleaved to yield 31-kDa N-terminal (N-ERC/mesothelin) and 40-kDa C-terminal (C-ERC/mesothelin) proteins. N-ERC/mesothelin is a soluble protein and has been reported to be a diagnostic serum marker of mesothelioma and ovarian cancer. Gastric cancer tissue also expresses C-ERC/mesothelin, but the significance of serum N-ERC levels for diagnosing gastric cancer has not yet been studied. We examined the latter issue in the present study as well as C-ERC/mesothelin expression in human gastric cancer tissues and cell lines. We immunohistochemically examined C-ERC/mesothelin expression in tissue samples from 50 cases of gastric cancer, and we also assessed the C-ERC/mesothelin expression in 6 gastric cancer cell lines (MKN-1, MKN-7, MKN-74, NUGC-3, NUGC-4 and TMK-1) using reverse transcription-polymerase chain reaction, flow cytometry, immunohistochemistry and immunoblotting. We also examined the N-ERC/mesothelin concentrations in the supernatants of cultured cells and in the sera of gastric cancer patients using an enzyme-linked immunosorbent assay (ELISA). N-ERC/mesothelin was detected in the supernatants of 3 gastric cancer cell lines (MKN-1, NUGC-4 and TMK-1) by ELISA, but its concentration in the sera of gastric cancer patients was almost same as that observed in the sera of the normal controls. In the gastric cancer tissues, C-ERC/mesothelin expression was associated with lymphatic invasion. N-ERC/mesothelin was secreted into the supernatants of gastric cancer cell lines, but does not appear to be a useful serum marker of gastric cancer.

## Introduction

Previously, we found that the *ERC* (expressed in renal carcinoma) gene was preferentially expressed in renal cancers in the Eker rat (1). Furthermore, we subsequently confirmed that ERC is a homolog of the human mesothelin gene, a gene that is strongly expressed in normal mesothelial cells, mesotheliomas, non-mucinous ovarian carcinomas and pancreatic ductal adenocarcinomas (2,3). The ERC/mesothelin gene (*MSLN*) encodes a 71-kDa precursor protein, which is cleaved by proteases to yield 31-kDa N-terminal (N-ERC/mesothelin) and 40-kDa C-terminal (C-ERC/mesothelin) proteins (4,5). N-ERC/mesothelin [also known as megakaryocyte-potentiating factor (MPF)] is a soluble protein and is released into the extracellular space and blood (4–9). C-ERC/mesothelin is a glycoprotein that is tethered to the cell surface by a glycosyl-phosphatidylinositol anchor.

In addition to mesothelioma, C-ERC/mesothelin is also expressed in ovarian cancer (4,10), pancreatic ductal adenocarcinoma (8,11,12), breast cancer (13), colorectal cancer (14) and esophageal adenocarcinoma (15). Several studies have detected C-ERC/mesothelin in human gastric cancer by immunohistochemical staining (16–18).

As for secreted N-ERC/mesothelin, we previously devised a novel enzyme-linked immunosorbent assay (ELISA) system for determining its concentration in serum and showed that it is useful for diagnosing human mesothelioma (7,9,19). However, its utility for diagnosing gastric cancer has not yet been examined. Therefore, in the present study we evaluated the expression and extracellular secretion of ERC/mesothelin in human gastric cancer tissues and cell lines derived from gastric cancer.

ELISA detected N-ERC/mesothelin in 3 of the 6 studied gastric cancer cell lines; however, its concentration in the sera of gastric cancer patients was almost the same as that in the sera of the normal controls. Therefore, N-ERC/mesothelin is not useful as a diagnostic marker of gastric cancer. Conversely, in the gastric cancer tissues, C-ERC/mesothelin expression was associated with lymphatic invasion. This result agrees with the findings reported by Einama *et al*(18).

## Materials and methods

### Cell lines

MKN-1 (adenosquamous carcinoma), MKN-7 (well-differentiated adenocarcinoma), MKN-74 (moderately differentiated adenocarcinoma) and NUGC-4 (signet ring cell carcinoma) cells, which are all derived from human gastric cancer, and Huh7 cells, which lack endogenous ERC/mesothelin expression and derived from human hepatocellular carcinoma, were provided by the RIKEN BRC Cell Bank (Tsukuba-shi, Ibaraki, Japan). TMK-1 (poorly differentiated adenocarcinoma) cells, which are derived from human gastric cancer, were provided by Hiroshima University (Hiroshima-shi, Hiroshima, Japan). NUGC-3 (poorly differentiated adenocarcinoma) cells, which are derived from human gastric cancer, were provided by the Health Science Research Resources Bank (Sennan-shi, Osaka, Japan). NCI-H226 cells, which were used as a positive-control and are derived from human mesothelioma, were provided by the American Type Culture Collection (Manassas, VA, USA). All of the cells, except the Huh7 cells, were cultured in RPMI-1640 medium supplemented with 10% fetal calf serum (FCS). The Huh7 cells were cultured in Dulbecco's modified Eagle's medium (DMEM) supplemented with 10% FCS. The culture supernatants and cells were harvested after being cultured for 48 h at 37°C under a 5% CO_2_ atmosphere, at which point they had reached >80% confluence.

### Reverse transcription-polymerase chain reaction (RT-PCR)

The mRNA levels of ERC/mesothelin in the cultured cells (MKN-1, MKN-7, MKN-74, NUGC-3, NUGC-4 and TMK-1) were analyzed by RT-PCR. RT-PCR was carried out using the Titan RT-PCR system (Roche Diagnostics GmbH, Mannheim, Germany) according to the manufacturer's instructions. Cells in Petri dishes were lysed with TRIzol reagent according to the acid guanidinium thiocyanate-phenol-chloroform extraction method (20) (Invitrogen Life Technologies, Carlsbad, CA, USA). Total RNA was extracted from these lysates according to the manufacturer's instructions. Total RNA (1 μg) was reverse transcribed for 30 min at 50°C and subjected to PCR amplification. The primers used to amplify ERC/mesothelin mRNA were as follows: sense, 5′-CAAGAAGTGGGAGCTGGAAG-3′ and antisense, 5′-GTCTCCAGGGACGTCACATT-3′. As a control for the RT-PCR, β-actin mRNA was amplified using the following β-actin-specific primers: sense, 5′-CCGCGAGAAG ATGACCCAGA-3′ and antisense, 5′-CAGGAGGAGCAATG ATCTTG-3′. All primers were purchased from Operon (Tokyo, Japan). After an initial denaturation step of 2 min at 94°C, each sample was subjected to 30 cycles of amplification (denaturation, 30 sec at 94°C; annealing, 30 sec at 55°C and elongation, 1 min at 68°C) followed by a final elongation step of 2 min at 68°C. The PCR products (20 μl) were analyzed on a 2% agarose gel containing 1 μg/ml ethidium bromide.

### Flow cytometric analysis

Cell surface C-ERC/mesothelin expression was analyzed by flow cytometry, as described previously (21). Briefly, 5×10^5^ cells were incubated for 30 min at 4°C with 1 μg/ml of the C-ERC/mesothelin-specific mouse monoclonal antibody 22A31, as described previously (21), or with 1 μg/ml of normal mouse IgG1κ diluted in 2% FCS (100 μl). After being washed with 2% FCS in medium, the cells were resuspended in 100 μl of 2% FCS in medium containing 2 μg/ml of Alexa Fluor 488-conjugated goat anti-mouse IgG (Molecular Probes, Eugene, OR, USA) to detect the primary antibodies, before being incubated at 4°C for 30 min. After being washed with phosphate-buffered saline (PBS), the stained cells were analyzed with an LSRFortessa™ cell analyzer (BD Biosciences, San Jose, CA, USA).

### Antibodies and western blot analysis

The culture supernatants of the MKN-1, MKN-7, MKN-74, NUGC-3, NUGC-4, and TMK-1 cells were harvested and adjusted with a solution containing 2% sodium dodecyl sulfate (SDS), 10% glycerol, 50 mM Tris-HCl (pH 6.8), and 100 mM dithiothreitol (DTT), before being boiled for 3 min. The culture supernatants were then electrophoresed on 10% Laemmli gels and transferred onto nitrocellulose membranes. The membranes were then blocked in a mixture of 1% skimmed milk and PBS supplemented with 0.1% Tween-20 (PBS-T) for 1 h at room temperature. Next, the membranes were incubated with the N-ERC/mesothelin-specific mouse monoclonal antibody (MoAb) 7E7, as described previously (7), or a rabbit anti-actin polyclonal antibody (sc-1616-R) (Santa Cruz Biotechnology, Inc., Santa Cruz, CA, USA; 1:500 dilution) in a mixture of PBS-T and 1% skimmed milk for 1 h at room temperature. The goat anti-mouse or anti-rabbit Ig secondary antibodies, which were conjugated with peroxidase labeled-dextran polymers (EnVision K4001 or K4003; DakoCytomation, Glostrup, Denmark) at a dilution of 1:50 in a mixture of PBS-T and 1% skimmed milk, were allowed to react with the membranes at room temperature for 1 h. The ECL detection system (GE Healthcare, Buckinghamshire, UK) was used to visualize ERC/mesothelin on the membranes.

### Human subjects

The present study included 50 patients with gastric cancer (40 men and 10 women; age range, 33–89 years; mean age, 71.5) who were diagnosed and provided blood samples between January 2009 and September 2011 at Juntendo Shizuoka Hospital, Juntendo University School of Medicine. All clinical diagnoses of gastric cancer were confirmed by microscopic examinations of the material obtained during surgery or endoscopic resection. The present study was approved by the Institutional Review Board of Juntendo Shizuoka Hospital, Juntendo University School of Medicine. All patients provided informed consent. Blood samples were collected from each patient before surgery or endoscopic resection. Thirty-seven healthy controls (age range, 53–79 years; mean age, 66.8) were sampled at random from a database, as described previously (9).

### Immunohistochemistry

Tissue sections (3 μm) were prepared from archival formalin-fixed, paraffin-embedded specimens. After being deparaffinized, the tissue sections were heated in target retrieval solution (DakoCytomation) for antigen retrieval and were then treated with 3% hydrogen peroxide. Non-specific binding sites were blocked by incubating the samples with 5% normal goat serum in PBS for 20 min at room temperature. Next, the sections were incubated overnight with primary antibody solutions diluted in PBS-T at 4°C. We used mouse monoclonal anti-human C-ERC/mesothelin antibody 22A31 (1:40 dilution) as the primary antibody and the EnVision+ system labeled with horseradish peroxidase (HRP) polymers (DakoCytomation) as the secondary antibody. Diaminobenzidine was used as the peroxidase substrate. The C-ERC/mesothelin immunostaining was analyzed using the scoring system described by Einama *et al*(18). For the immunostained slides, the proportion of stained cancer cells was scored as +1 for 1–10, +2 for >10–50 and +3 for >50%. The intensity of the staining was scored as +1 for weak intensity and +2 for moderate to strong intensity, and the location of the staining was also recorded; i.e., in the luminal membrane or the cytoplasm. The final evaluation of C-ERC/mesothelin expression was based on the following scoring system: ‘positive-staining’ was defined as a proportion score of ≥+3 and/or an intensity score of +2, while ‘negative staining’ was defined as a total score of <+3, except in cases involving a proportion score of +1 and an intensity score of +2.

### Sandwich ELISA

The N-ERC/mesothelin concentrations in the patient sera and the cell culture supernatants of the MKN-1, MKN-7, MKN-74, NUGC-3, NUGC-4 and TMK-1 cells were analyzed using the sandwich ELISA method (7), which was performed as described previously (7,9) using the 7E7 MoAb and the HRP-conjugated polyclonal antibody-282. The absorbance of the solution was measured at 450 nm in an ELISA reader (EMax; Molecular Devices, Sunnyvale, CA, USA).

### Statistical analysis

The data were analyzed with GraphPad Prism 5.0 (GraphPad Software, San Diego, CA, USA). The measurement data were analyzed using the Mann-Whitney and Kruskal-Wallis tests, while the categorical data were analyzed using Fisher's exact test. P<0.05 was considered to indicate a statistically significant result.

## Results

### Expression of ERC/mesothelin mRNA in human gastric cancer cell lines

**Figure 1:**
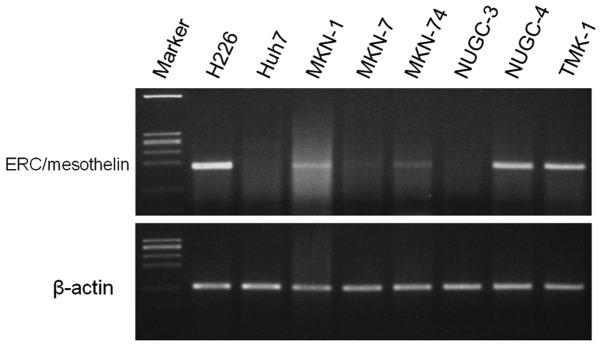
ERC/mesothelin transcript was detected in human gastric cancer cell lines by RT-PCR. H226 cells derived from human mesothelioma were used as a positive control. Huh7 cells derived from human hepatocellular carcinoma were used as a negative control. The other cell lines are derived from human gastric cancer.

**Figure 2:**
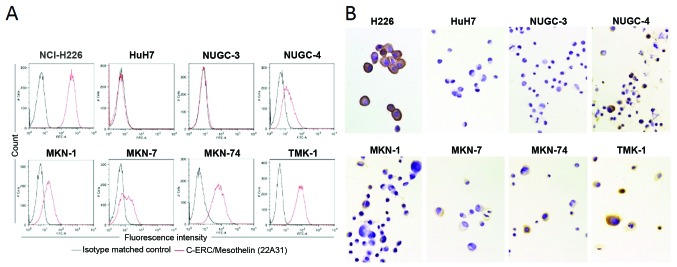
C-ERC/mesothelin expression. (A) Cell surface C-ERC/mesothelin expression in the human gastric cancer cell lines as determined by flow cytometry. (B) Immunohistochemical staining of C-ERC/mesothelin in the human gastric cancer cell lines using 22A31 antibody. Magnification, ×400.

**Figure 3:**
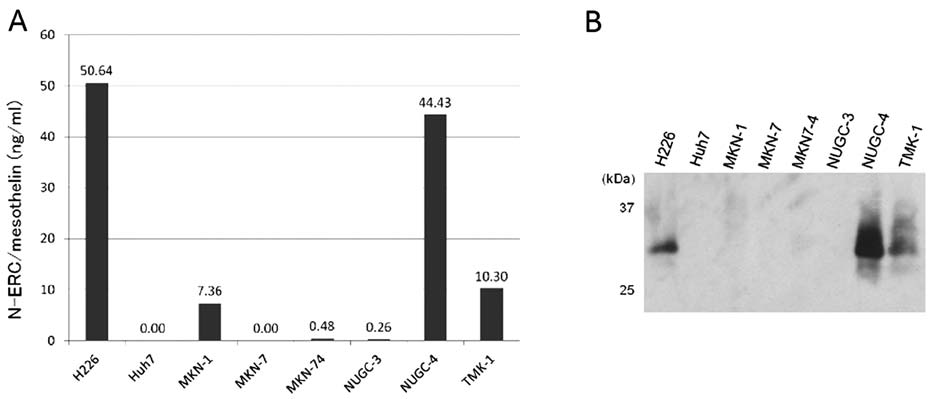
ERC/mesothelin expression in human gastric cancer cell lines. (A) ELISA detected N-ERC/mesothelin that had been secreted into the cell culture media of human gastric cancer cell lines. (B) Immunoblotting detected N-ERC/mesothelin (31 kDa) that had been secreted into the cell culture media of human gastric cancer cell lines. ELISA, enzyme-linked immunosorbent assay.

In 5 of the 6 investigated gastric cancer cell lines (MKN-1, MKN-7, MKN-74, NUGC-4 and TMK-1), ERC/mesothelin mRNA and C-ERC protein were detected by RT-PCR (Fig. 1), flow cytometry (Fig. 2A) and immunohistochemistry (Fig. 2B). The C-ERC/mesothelin was localized in the cytoplasm and/or on the cell membrane (Fig. 2). ELISA demonstrated that 3 of these 5 cell lines (MKN-1, NUGC-4 and TMK-1) excreted N-ERC into the culture medium (Fig. 3A). However, western blotting only detected N-ERC in the media from 2 cell lines (NUGC-4 and TMK-1) (Fig. 3B).

### C-ERC/mesothelin expression in human gastric cancer

**Figure 4:**
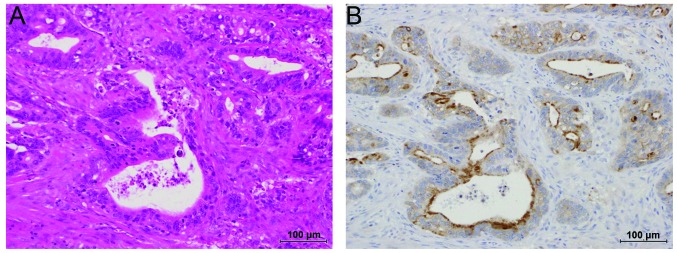
Immunohistochemical analysis. (A) Hematoxylin and eosin staining of well-differentiated tubular adenocarcinoma cells. (B) Strongly positive C-ERC/mesothelin staining. Scale bar, 100 μm. A and B are serial sections.

Immunohistochemically, positive C-ERC/mesothelin staining was observed on the cancer cell membrane and in the cytoplasm (Fig. 4). Among the 50 tumors examined, 29 (58%) were positive for C-ERC/mesothelin. Luminal membrane expression of C-ERC/mesothelin was observed in 15 cases (30%).

### Clinicopathological correlations

The correlations between the clinicopathological features and C-ERC/mesothelin expression in the primary tumors are summarized in Table I. C-ERC/mesothelin positivity was not associated with any of the parameters. However, the incidence of C-ERC/mesothelin luminal membrane expression was correlated with lymphatic invasion (P=0.0286).

### Serum levels of N-ERC/mesothelin in the gastric cancer patients

**Figure 5:**
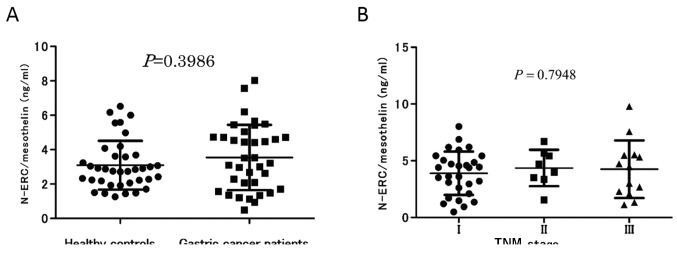
Serum N-ERC/mesothelin concentrations in the gastric cancer patients. (A) No significant difference was noted in the serum N-ERC/mesothelin concentrations between cancer patients and healthy controls. (B) When TNM stage was analyzed, no correlation was noted with the serum N-ERC/mesothelin concentration.

The N-ERC/mesothelin levels in the sera of the patients were evaluated. We selected 38 of the 50 patients for age-matching with the control group. The sera of the 38 age-matched gastric cancer patients (26 men and 10 women, age range, 33–78 years; mean age, 65.0) were compared with those of the 37 healthy controls (24 men and 13 women, age range, 53–79 years; mean age, 66.8) as described previously (9). There was no significant difference between the serum N-ERC/mesothelin concentrations of the cancer patients and healthy controls (P=0.3986) (Fig. 5A). When TNM stage was analyzed, a significant correlation with the serum N-ERC/mesothelin concentration was not detected (P=0.7948) (Fig. 5B).

## Discussion

We evaluated the expression and extracellular secretion of ERC/mesothelin in human gastric cancer cell lines. All of the human gastric cancer cell lines, except NUGC-3, expressed ERC/mesothelin mRNA and C-ERC/mesothelin protein. However, their expression levels in the human gastric cancer cells were lower than those noted in the H226 human mesothelioma cells (Figs. 1 and 2). The ERC/mesothelin mRNA levels detected in the cultured cells were approximately correlated with the expression level of C-ERC/mesothelin protein, suggesting that this gene was mainly regulated at the transcriptional level.

ELISA (7,9) detected N-ERC/mesothelin in the supernatants of 3 cell lines (MKN-1, NUGC-4 and TMK-1). The N-ERC/mesothelin concentration in the culture medium roughly reflected the mRNA levels detected in the cells (Figs. 1 and 3). Immunoblotting only detected secretory N-ERC/mesothelin in 2 cell lines (NUGC-4 and TMK-1), indicating that the ELISA was more sensitive than our immunoblotting technique for detecting N-ERC/mesothelin.

As it was demonstrated that the gastric cancer cells excrete N-ERC/mesothelin into the culture medium, we attempted to detect it in the sera of the gastric cancer patients and explored the possibility of using it as a diagnostic tool. First, we compared the serum N-ERC/mesothelin levels of all 50 patients (age range, 33–89 years, mean age, 71.5) with those of the 102 healthy controls (age range, 30–79 years, mean age, 52.3), which were selected from a database as described previously (9). As a result, we found that the 50 patients exhibited a significantly higher serum N-ERC/mesothelin level than that in the control group (P<0.0001). However, when we randomly selected 38 patients with gastric cancer and 37 age-matched healthy controls, there was no significant difference between the mean serum N-ERC/mesothelin concentration of the cancer patients and that of the healthy control group. The serum level of N-ERC/mesothelin is reported to increase with age (9), and our initial analysis without age-matching produced biased results since the gastric cancer patients tended to be older than the healthy controls.

In the present study, the serum N-ERC/mesothelin concentration was not found to be a useful diagnostic marker of gastric cancer. Previously, we and others have shown that the serum N-ERC/mesothelin level is useful for diagnosing human mesothelioma and ovarian cancer (7,9,19,22,23). Conversely, in a previous study we did not find any significant difference in the serum concentration of N-ERC/mesothelin between pancreatic ductal carcinoma patients and healthy controls (8). Sharon *et al*(24) also reported that neither the serum concentration of mesothelin nor that of MPF was elevated in patients with pancreatic or biliary cancer. At present, we cannot explain why N-ERC/mesothelin is abundantly excreted into the serum in some malignancies but not in others, despite the fact that C-ERC/mesothelin is expressed in all of these malignancies.

Creaney *et al*(25) demonstrated that the mesothelin levels in patients with malignant pleural mesothelioma were strongly correlated with their tumor burdens. Gastric tumors are usually smaller than mesotheliomas, and this difference in tumor mass may explain why patients with mesothelioma exhibit increased serum N-ERC/mesothelin levels, but gastric cancer patients do not. It is also possible that the endogenous level of ERC/mesothelin expression is higher in mesothelioma cells than in gastric cancer cells, as shown in Figs. 1 and 2 (H226 mesothelioma cells vs. other gastric cancer cells). Other factors such as protease activity may also be responsible for the lower serum N-ERC/mesothelin levels experienced in gastric cancer cases.

We also examined the expression of C-ERC/mesothelin in cultured gastric cancer cell lines and human gastric cancer tissues by immunohistochemical staining. In the present study, C-ERC/mesothelin positivity was detected in 5 of 6 gastric cancer cell lines (83.3%). In addition, C-ERC/mesothelin positivity was observed in human gastric cancer tissues from 29 of the 50 cases (58%). These results concur with those reported by Scholler *et al*(16), Baba *et al*(17) and Einama *et al*(18). Two of these 3 studies examined the clinicopathological features and C-ERC/mesothelin expression in gastric cancer (17,18). Baba *et al*(17) reported that C-ERC/mesothelin expression appears to be correlated with prolonged patient survival. Conversely, Einama *et al*(18) reported that mesothelin expression was not correlated with the overall survival of gastric cancer patients. However, they argued that luminal membrane C-ERC/mesothelin expression was associated with lymphatic invasion and poor patient outcomes (18). In the present study, C-ERC/mesothelin positivity was not associated with any parameters. However, it was demonstrated that the incidence of C-ERC/mesothelin luminal membrane expression was correlated with lymphatic invasion (P=0.0286). This finding is consistent with the results reported by Einama *et al*(18) and agrees with previous studies that showed that ERC/mesothelin enhances cell adhesion and invasion (4). In an *in vitro* study, Kawamata *et al*(14) demonstrated that C-ERC/mesothelin provokes the lymphatic invasion of colorectal adenocarcinoma cells.

In conclusion, N-ERC/mesothelin was secreted into the culture supernatants of gastric cancer cell lines; however, increased serum N-ERC/mesothelin concentrations were not specific to gastric cancer patients. Therefore, N-ERC/mesothelin does not appear to be useful as a serum marker of gastric cancer. The present study is small and requires further validation, and did not include cases involving the peritoneal dissemination of cancer cells. ERC/mesothelin is endogenously expressed in the peritoneal mesothelium, and future studies should examine whether the serum N-ERC/mesothelin level could be used as a marker of peritoneal invasion by gastric cancer.

## Figures and Tables

**Table I:** Clinicopathological characteristics of the gastric cancer patients according to C-ERC/mesothelin staining.

	C-ERC expression			Luminal membrane expression		
				
Parameter	Positive (n=29)	Negative (n=21)	P-value	Positive (n=15)	Negative (n=35)	P-value
Gender			0.3145			0.3044
Male	24	14		13	25	
Female	5	7		2	10	
Age, years^a^	73.17±10.52	69.24±13.40	0.3757	72.27±12.20	71.2±11.87	0.8404
Tumor size, mm^a^	51.90±51.62	40.90±30.75	0.6021	48.87±29.30	46.60±49.38	0.433
Histology			0.1226			0.1764
Well-moderate	23	12		13	22	
Poor	6	9		2	13	
T classification			0.569			0.5355
1	15	13		7	21	
2–4	14	8		8	14	
N classification			1			0.5279
0	18	13		8	23	
1–3	11	8		7	12	
Lymphatic invasion			0.0848			0.0286^b^
Positive	19	8		12	15	
Negative	10	13		3	20	
Venous invasion			0.7685			0.5279
Positive	12	7		7	12	
Negative	17	14		8	23	
TNM stage			0.7734			0.3557
1	16	13		7	22	
2–4	13	8		8	13	

a Values are expressed as mean ± SD.

b Statistically significant at p<0.05.
